# Tuberculous Osteomyelitis of Calcaneum: A Rare Case

**DOI:** 10.7759/cureus.70659

**Published:** 2024-10-01

**Authors:** Vivek H Jadawala, Sanjay V Deshpande, Ankur Salwan, Hitendra Wamborikar, Rohit Randad, Rushi Jadawala

**Affiliations:** 1 Department of Orthopaedics, Jawaharlal Nehru Medical College, Datta Meghe Institute of Higher Education and Research, Wardha, IND; 2 Department of Medicine, DY Patil Medical College, Navi Mumbai, IND

**Keywords:** calcaneum, extrapulmonary manifestations of tuberculosis, extrapulmonary tuberculosis, infection, osteoarticular tuberculosis, osteomyelitis, tb, tb osteomyelitis, tuberculosis

## Abstract

Tuberculosis (TB) is a major public health challenge in rapidly developing economies. TB commonly affects the lungs but, in rare cases, can spread to other organs such as intestines, bones, and lymph nodes. Tuberculous osteomyelitis (OM) of calcaneum in adults is rarely encountered and not very commonly reported in the literature. Most of the cases are either misdiagnosed or treating physicians often face significant delays in diagnosing Tuberculous OM of the calcaneum. We report the case of a female in her mid-thirties who had a known case of pulmonary TB for the past 10 years with tuberculous OM of the left calcaneum for three years. Initially, she was able to bear weight over her heel and walk normally, but for the past year, she was unable to bear weight over her left heel and walked with toe tips. Although being a known case of TB, the patient gave no history of any significant weight loss, even a rise in temperatures, or any systemic complaints. Tubercular OM of the calcaneum is an extremely rare clinical entity, which commonly leads to delay in diagnosis and further delay in therapeutic intervention. Patients who present with chronic osteo-articular lytic infections with past history of TB should be suspected of having osteo-articular infection with tuberculous bacilli and must be evaluated. Based on the current case scenario, the tubercular OM of the calcaneum should be diagnosed early and prompt treatment in the form of anti-tubercular therapy (AKT) should be initiated. Most cases respond well to conservative treatment in a few weeks after starting AKT. Surgical intervention should be reserved in cases refractory to conservative management or those with extensive soft tissue or joint involvement with discharging sinuses. In such cases, sequestrectomy and curettage with surgical excision of the sinus tract are needed.

## Introduction

Tuberculosis (TB) is a major public healthcare concern in rapidly developing countries such as India. It is one of the leading causes of death globally and in India and kills approximately 2.2 lakh people annually [1]. TB is an infectious disease caused by Mycobacterium tuberculosis bacteria. It is transmitted through the air via droplets that come out from the throat and lungs of a person with active tuberculosis infection when they sneeze, cough, speak, or sing [2]. India has the highest burden of TB with about two deaths occurring per three minutes [2]. Osteoarticular tuberculosis is diagnosed in approximately 1.5-2.5% of all tuberculosis cases [3]. TB of ankle and foot (AFTB) comprises about 10-15% of all osteo-articular tuberculosis, and the majority of these patients do not show any signs of pulmonary involvement [4]. Moreover, there are only limited studies and cases reported on tuberculous osteomyelitis of calcaneum in adults.

In most cases, there is often a delay in the diagnosis of tuberculous osteomyelitis of calcaneum due to a lack of awareness of the treating doctor. In cases suspected of having tuberculous osteomyelitis of the calcaneum, prompt diagnosis and early treatment of the infection is very important to limit the further spread of the infection. Here, we report the case of a female in her mid-thirties who had a known case of pulmonary tuberculosis for the past 10 years with tuberculous osteomyelitis of the left calcaneum for three years.

## Case presentation

A 35-year-old lady presents to the orthopaedics outpatient department (OPD) at our institute with dull-aching pain and swelling over her left heel for the last three years with no history of any trauma or falls. Initially, she was able to bear weight over her heel and walk normally, but for the past year, she was unable to bear weight over her left heel and walked on toe tips. On detailed history taking, she gives a history of being diagnosed with pulmonary Koch’s about 10 years ago, for which she took anti-tubercular drugs for a few weeks but did not complete the course of her anti-tubercular treatment. Although being a known case of TB, the patient gave no history of any significant weight loss, evening rise of temperatures, or any systemic complaints. There was no history of any loss of appetite.

**Figure 1:**
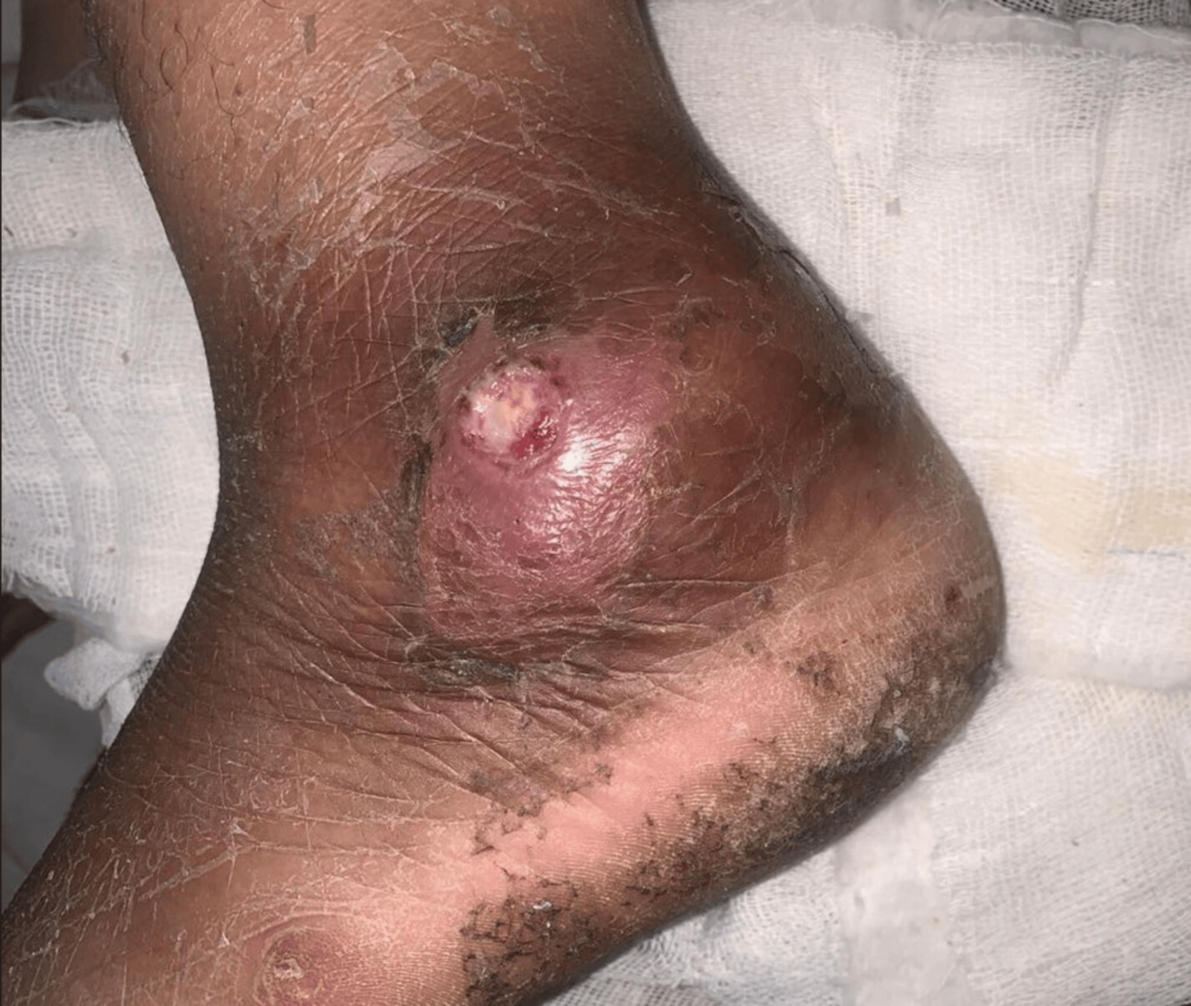
Clinical image of the wound (size 1.5 x 1.5 cm) over the lateral aspect of the left ankle

On clinical examination of the left ankle and foot, there was minimal swelling over the posterior aspect of the heel and a small circular wound of size 1.5 cm radius with discharging sinus over the lateral aspect of the ankle just below the lateral malleolus, as shown in Figure 1. On palpation, there was minimal local rise of temperature over the posterior and lateral aspects of the heel and ankle. Tenderness to deep palpation was present over the calcaneum. The overlying skin was tense and inflamed with active pus discharge from the wound. Popliteal as well as inguinal lymph nodes were not palpable.

**Figure 2:**
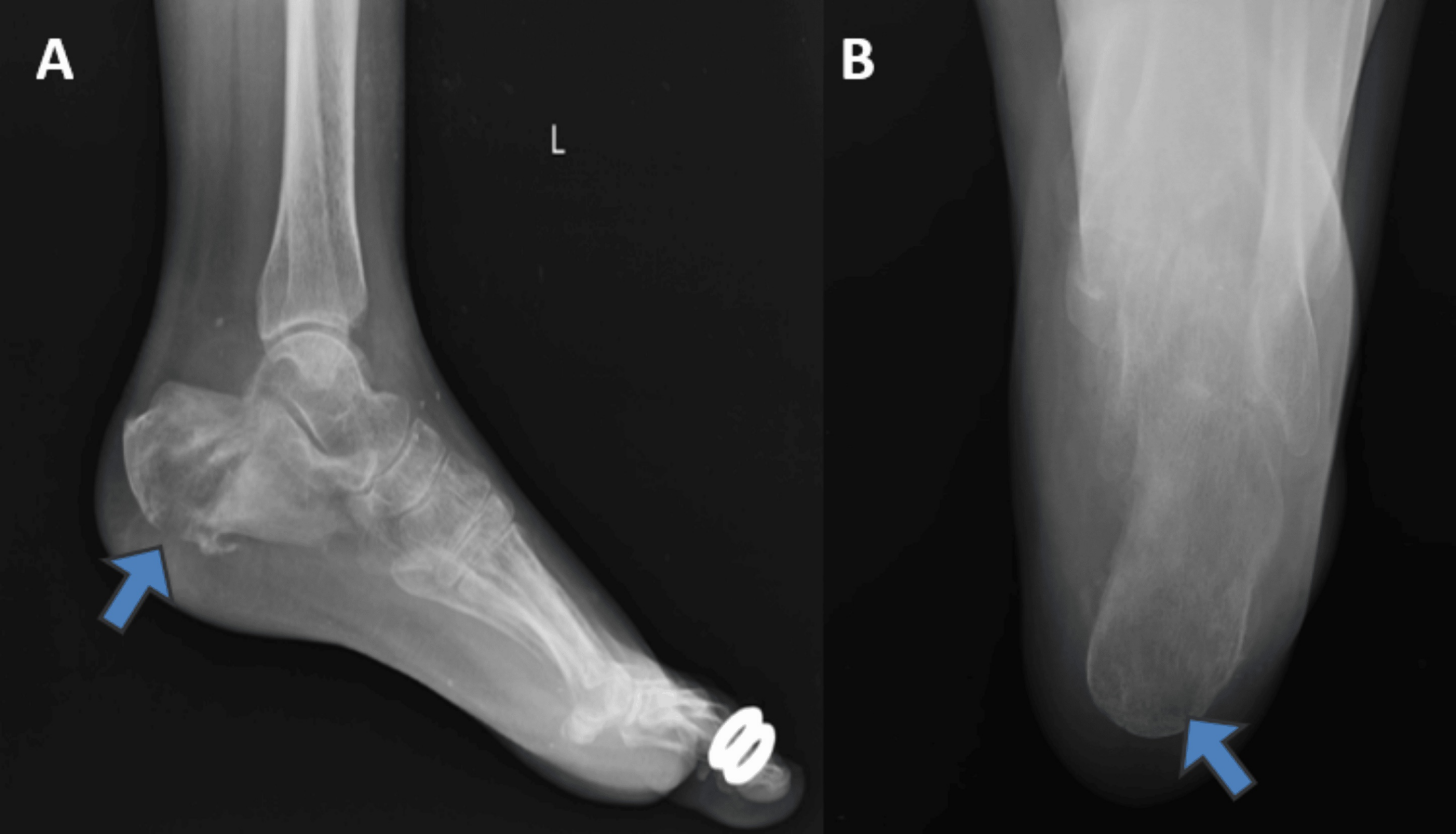
Plain radiograph of the left ankle with the foot A – Lateral view showing evidence of osteolytic lesion in the body of the calcaneum with cortical defect over the planter aspect of the calcaneum posteriorly (blue arrow) B – Axial view of the calcaneum with osteolysis over the posterior aspect and body of the calcaneum (blue arrow)

Radiographs of the left ankle (lateral) and calcaneum axial view were performed, which showed evidence of osteolytic lesion in the body of the calcaneum with cortical defect over the plantar aspect of the calcaneum posteriorly, as shown in Figure 2.

**Figure 3:**
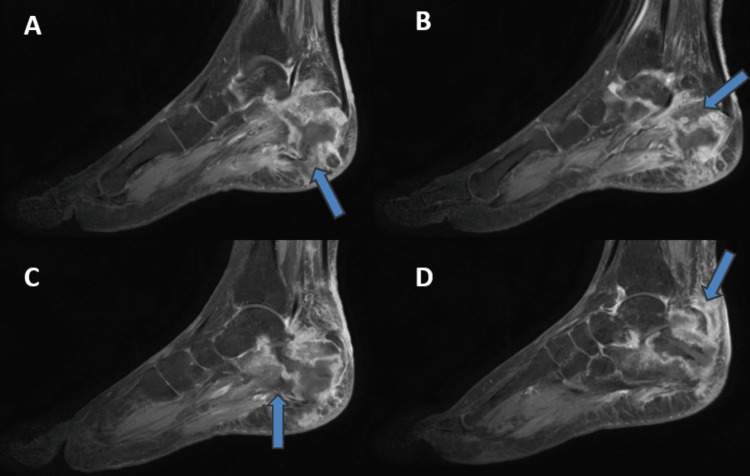
Magnetic resonance imaging with contrast of left ankle – sagittal section A to D - Medial to lateral - showing marrow edema with signal intensity changes in the calcaneum. Image A - Infective focus in the calcaneum body with abscess formation (blue arrow) Images B and C showing bony destruction with involvement of the subtalar and ankle joint (blue arrow) Image D showing the postero-lateral extension of the abscess with sinus tract formation (blue arrow)

**Figure 4:**
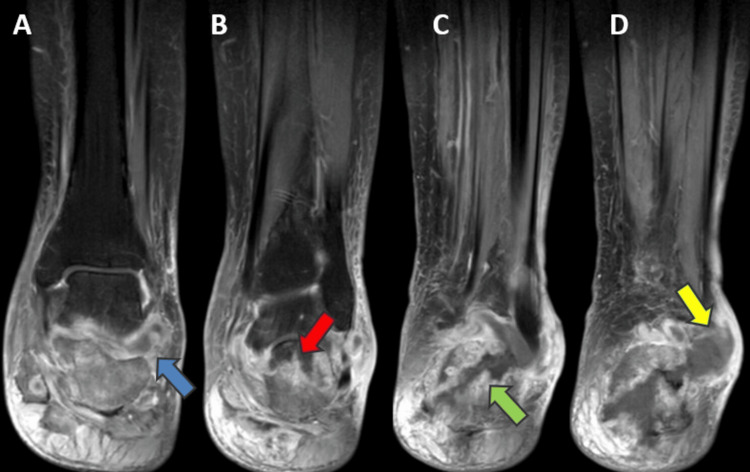
Magnetic resonance imaging with contrast of the left ankle – coronal section A to D - Anterior to posterior - showing marrow edema and signal intensity changes in the calcaneum and ankle joint Image A - Showing extra-articular extension of the abscess in the area surrounding the ankle joint Image B - Showing the pus-filled area in the body of the calcaneum Image C and D - Showing extensive sequestrum formation in the calcaneum with sinus tract extending laterally

Magnetic resonance imaging (MRI) with contrast of the left ankle was performed, which showed a pathological fracture of the posterior aspect of the calcaneum body. A contrast-enhanced scan showed diffuse marrow oedema with peripherally enhancing collection measuring approximately 3.5 cm x 3 cm in the posterior third of the calcaneum suggestive of abscess formation. There is the presence of another peripherally enhancing collection measuring approximately 4 cm x 2.8 cm seen in the soft tissue, just lateral to the calcaneum and posterior compartment of the ankle joint suggestive of abscess formation with presence of granulation tissue. The MRI scan also showed gross soft tissue oedema around the ankle joint with the presence of mild ankle joint and subtalar joint effusion (Figure 3 and Figure 4). Laboratory investigations were performed, which showed elevated levels of erythrocyte sedimentation rate (ESR) and raised C-reactive protein (CRP). Lymphocytosis with elevated white blood cell (WBC) count was seen, as shown in Table 1. A chest radiograph was performed, which showed the presence of fibrotic consolidation in the middle and lower lobes of the right lung and lower lobe of the left lung with hilar adenopathy bilaterally.

**Table 1:** Inflammatory laboratory profile charting of the patient with normal values ESR - Erythrocyte sedimentation rate; CRP - C-reactive protein; WBC - White blood cell count

	ESR	CRP	WBC count
Normal Range	0 to 30 mm/hr	0.3–1.0 mg/dL	4,500–11,000 cells per microliter
Patient's Laboratory Values			
On Admission	65 mm/hr	6.6 mg/dL	19600 cells per microliter
Post-operative day 1	82 mm/hr	4.2 mg/dL	13100 cells per microliter
Post-operative day 7	54 mm/hr	2.1 mg/dL	10400 cells per microliter
Post-operative day 12	40 mm/hr	1.4 mg/dL	9900 cells per microliter

**Figure 5:**
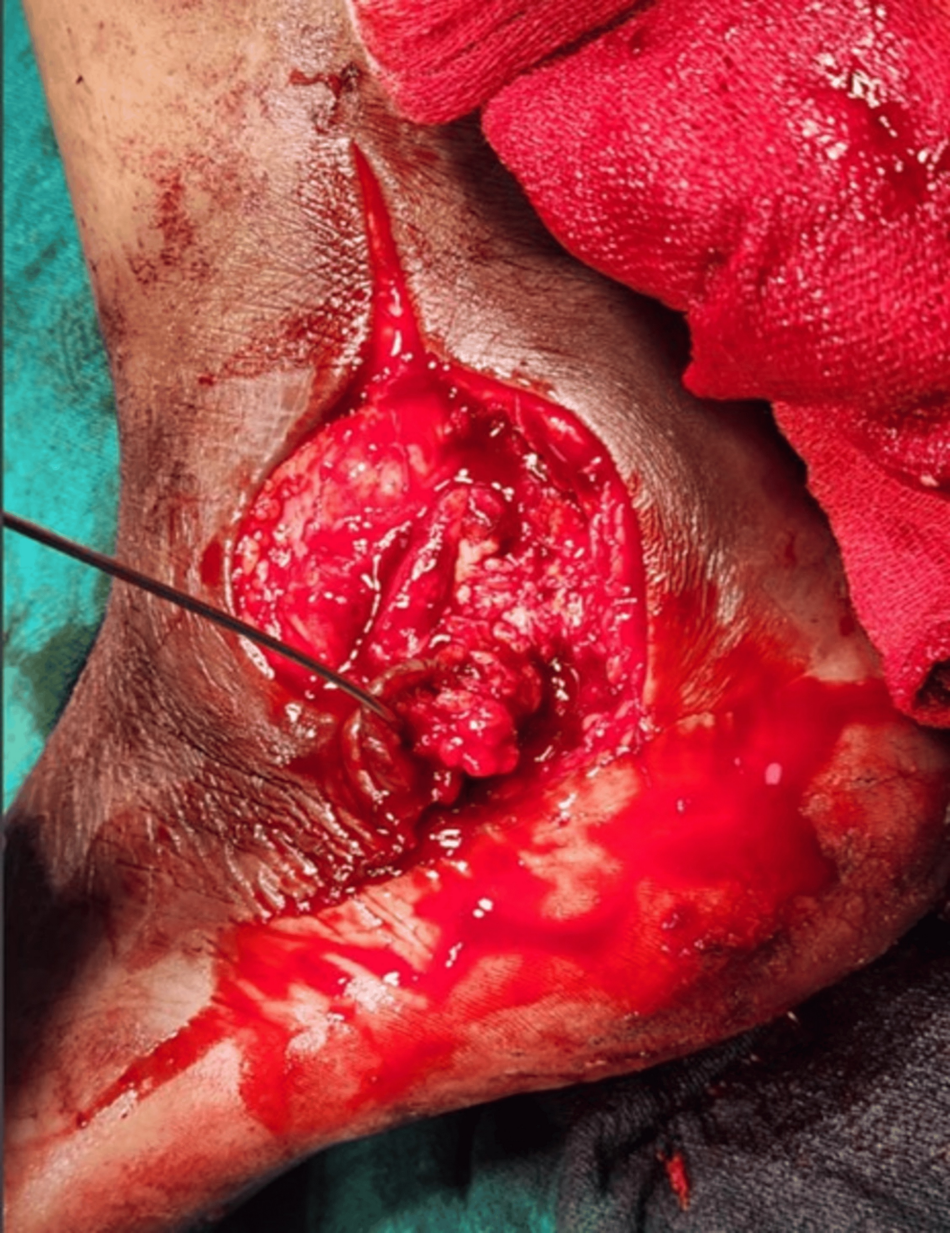
Intra-operative clinical image showing the postero-lateral approach to the ankle and calcaneum with abscess

**Figure 6:**
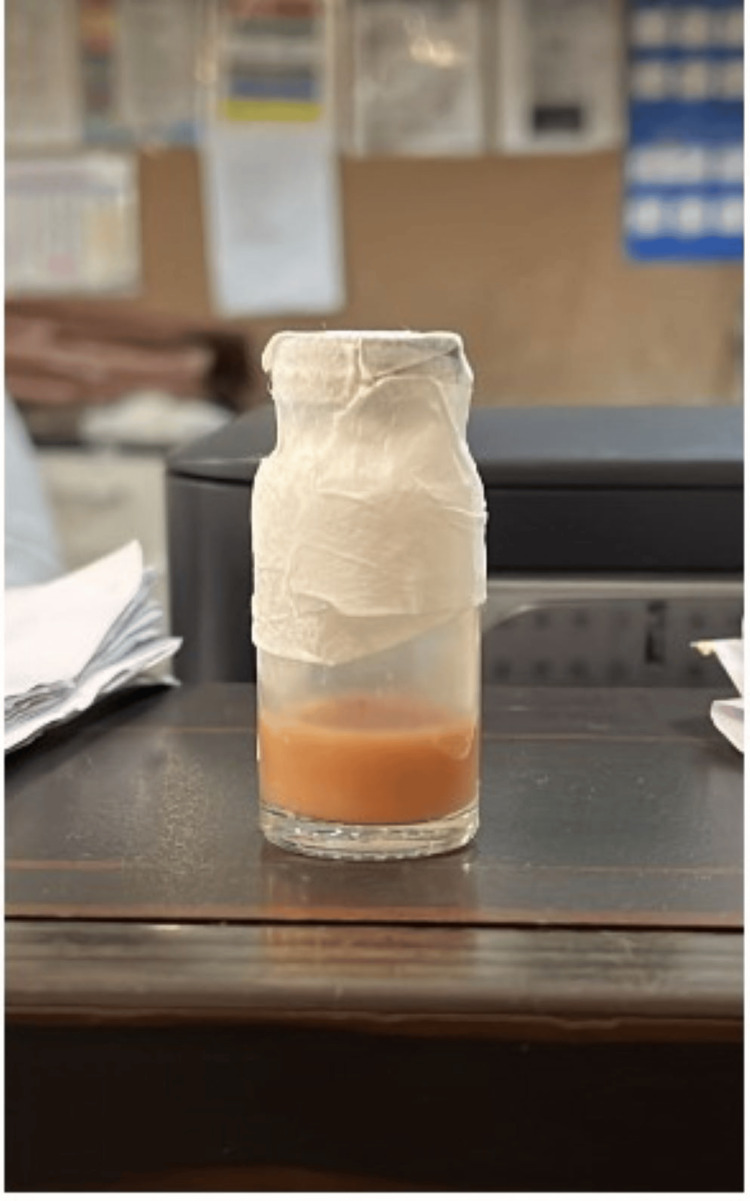
Sample collected from the abscess showing caseating creamy whitish-yellow pus

The patient was investigated further for TB as she was suspected of having tuberculous osteomyelitis of the left calcaneum and ankle. The patient has explained the possibility of TB infection, and after proper counseling, her sputum sample was collected and sent for an acid-fast bacillus (AFB) smear test. The patient was planned for incisional biopsy and excision of the sinus tract along with curettage for calcaneal osteomyelitis. With proper consent and fitness from anaesthetist, the patient was taken to the operating room. Under all aseptic precautions, a postero-lateral approach to the ankle was taken, and the calcaneum was exposed laterally (Figure 5). Intra-operatively, lytic areas around the posterior-lateral part of the calcaneum were present along with the presence of an abscess cavity in the retrocalcaneal bursae. The sinus tract was excised and granulation tissue along with abscess was drained and collected. The collected sample was caseous with a creamy whitish-yellow colour (Figure 6). All the dead, necrotic tissues were removed, and thorough lavage was given with betadine, hydrogen peroxide, and normal saline. Curettage and sequestrectomy of calcaneum osteomyelitis were done. A thorough wash was given with 6 L of normal saline, and closure was done in layers. Skin closure was not possible due to poor wound site following sinus tract excision. The patient was then started with platelet-rich plasma infiltration at the wound edges for wound healing. Samples collected were sent for histopathology and for cartridge-based nucleic acid amplification test (CB-NAAT).

**Figure 7:**
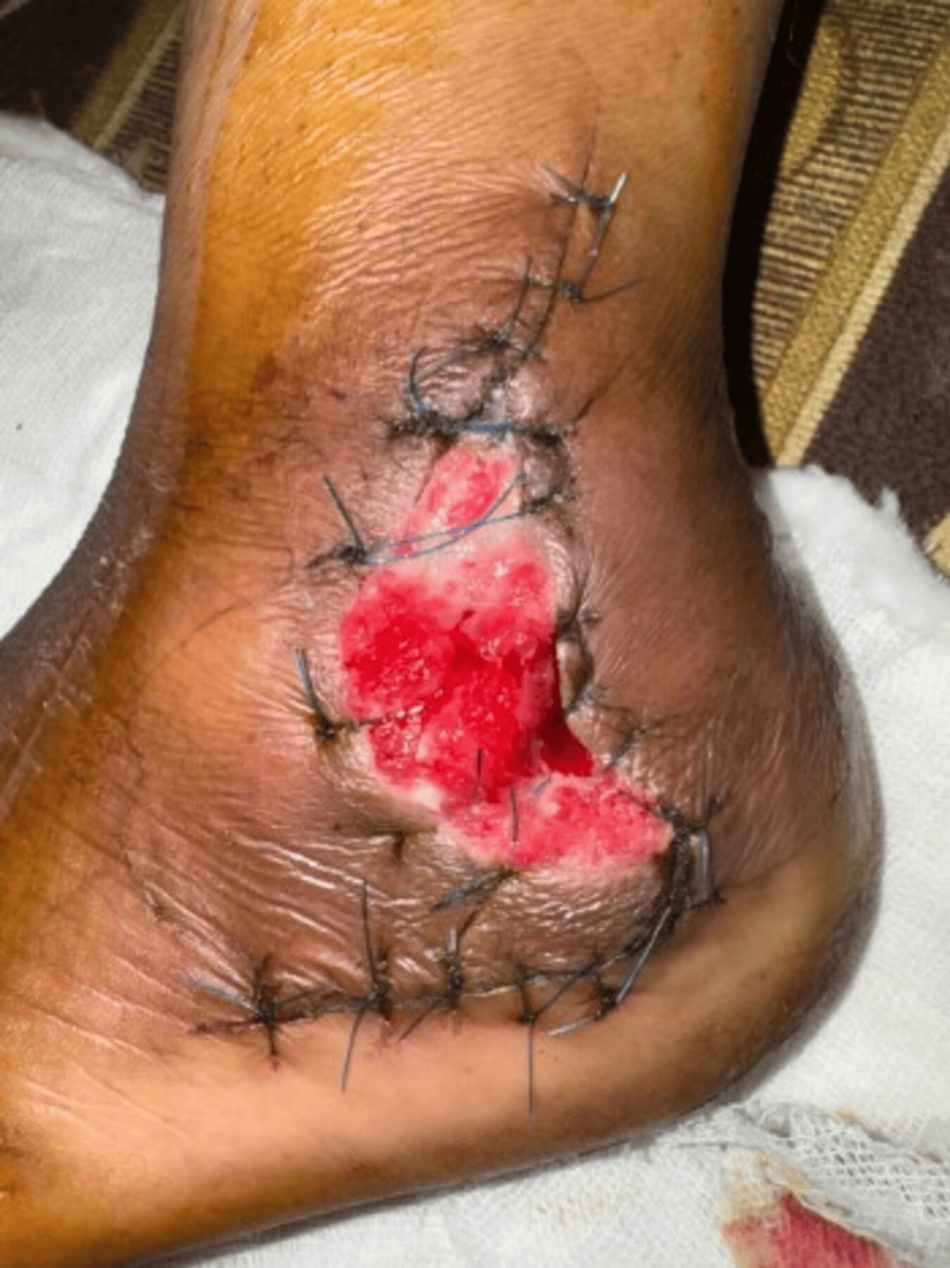
Post-operative clinical image on day 10 following surgery showing healing wound with the presence of healthy granulation tissue

Post-operatively, the patient was given below knee posterior slab and advised strict non-weight bearing for six weeks. Post-operative dressings along with PRP infiltration at wound edges were done on day two, day five, and day 10 (Figure 7). Suture removal was done on day 13. The patient was discharged on day 14 and advised to follow up in OPD for further PRP infiltration for wound healing.

**Figure 8:**
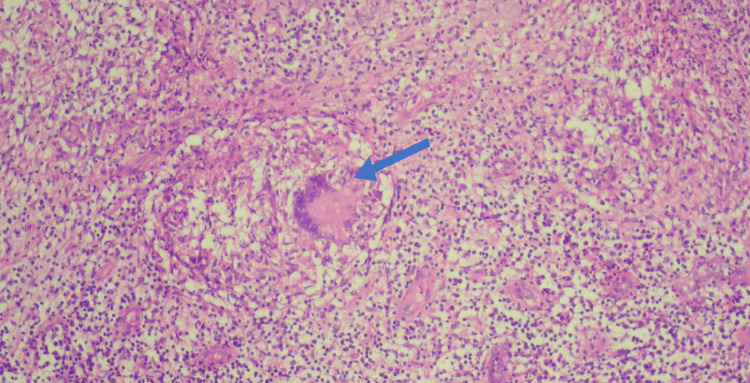
Histopathological examination (H&E stained, 100X) showing granuloma formation with caseous necrosis with Llanghan’s giant cells (blue arrow)

Although sputum for AFB was negative, CB-NAAT, which is highly sensitive for the diagnosis of TB infection, was positive for Mycobacterium tuberculosis. No drug resistance was noted in the CB-NAAT report. The histopathology report also showed granuloma formation with caseous necrosis and Langhan’s giant cells in the collected sample (Figure 8). The patient was then counseled and started on anti-tubercular therapy under directly observed treatment, short course (DOTS) therapy.

## Discussion

Tuberculous osteomyelitis of the calcaneum is very rare and quite uncommon in adults [5,6]. Due to uncommon location, rarity, and unawareness regarding AFTB, there is a delay in diagnosis and treatment in most cases. At the time of diagnosis, the infection commonly reaches its fulminant destructive stage. Martini et al. [7] provided the classification for foot and ankle tuberculosis (Table 2).

**Table 2:** Classification for foot and ankle tuberculosis (AFTB) Martini et al. [7]

Stage	Feature
1	Localized osteoporosis with No evidence of significant bony changes
2	Cavity formation or significant bony destruction with lytic areas in the bone
3	Complete destruction of the involved bone without nearby joint involvement
4	Massive destruction with joint involvement

Based on the above classification (Table 2), the patient in the present case report corresponds to Stage 4 of Martini et al.'s classification of foot and ankle TB with massive destruction of the calcaneum along with involvement of sub-talar and ankle joint as well as surrounding soft tissue involvement with abscess formation.

Tubercular OM of calcaneum, if not diagnosed and left untreated, may lead to serious functional disability with involvement of the ankle and subtalar joint [8]. Patients with osteoarticular infection with a suspected or past history of tuberculosis should always be evaluated by chest radiographs for primary foci in the lungs [9]. Plain radiographs of the foot and ankle might not show bony destruction or tubercular lesions early in the disease. By the time the radiographic involvement of the bone is seen, the infection would have already spread and advanced to distant sites [10]. CT scans show the presence of sequestrum and involucrum early in the disease process than plain radiographs and are often necessitated as a guide for planning biopsy. MRI of the involved region shows altered signal intensity on both T1-weighted and T2-weighted scans. Marrow edema is a common finding and corresponds to the initiation of the destructive phase of bony involvement of tuberculosis. Local spread to surrounding soft tissues and joints can also be identified and staged.

Laboratory investigations, such as ESR and CRP, although suggestive of infective etiology, are not specific to tuberculosis. Biopsy with culture and histopathological examination helps in diagnosing most cases of osteoarticular tuberculosis [11]. CB-NAAT is the gold standard test useful for the diagnosis of extra-pulmonary tuberculous infections, whereas, in cases with paucibacillary infection, acid-fast Mycobacterium tuberculosis might not be isolated on Ziehl-Neelsen (ZN) staining [12]. Histopathological examination may show characteristic central caseous necrosis with epitheloid granuloma formation and langhan's giant cells [13].

Anti-tuberculosis chemotherapy is the primary treatment for tubercular OM of calcaneum as well as other osteoarticular tuberculosis. Diagnosis should be made as early as possible so as to start prompt and early treatment to avoid functional disability in patients with osteoarticular tuberculosis. Directly observed treatment, short course (DOTS) therapy for at least 12 months is the standard of care in most countries when it comes to osteoarticular tuberculosis [14]. Surgical treatment for tubercular calcaneal OM is indicated in cases with large abscess formation with extensive soft tissue and joint involvement. In such cases, sequestrectomy and curettage along with biopsy of the collected specimen and excision of sinus tracts may be necessitated. Other indications for surgical interventions may include cases that are resistant to chemotherapy, healed cases of osteoarticular tuberculosis with residual pain, deformity, or joint contractures. In these cases, reconstruction of joints for deformity correction with functional reversal is the primary goal of treatment [15].

In some cases of osteoarticular tuberculosis with extensive soft tissue damage such as, in the current case, soft tissue coverage by the skin may not be possible following surgical intervention. In those cases, other modalities of treatment for adequate soft tissue cover may be needed such as skin grafting, vacuum-assisted closure (VAC) dressings using negative pressure wound therapy (NPWT), flap reconstructions, and platelet-rich plasma infiltration [16-18].

## Conclusions

Tubercular OM of the calcaneum is an extremely rare clinical entity that commonly leads to misdiagnosis and delays in therapeutic intervention. Suspected cases or cases with a past history of tuberculosis with long-standing osteoarticular lytic infections should be suspected of having extrapulmonary tuberculosis in the bone and must be evaluated. Based on the current case scenario, we conclude that tubercular OM of the calcaneum should be diagnosed early and prompt treatment in the form of anti-tubercular therapy (AKT) should be initiated. Most cases respond well to conservative treatment in a few weeks after starting AKT. Surgical intervention should be reserved in cases refractory to conservative management, or those with extensive soft tissue or joint involvement with discharging sinuses. In such cases, sequestrectomy and curettage with surgical excision of the sinus tract are needed. Joint reconstructive procedures should be kept reserved for cases with healed tubercular infections with residual deformity.
